# A Literature Review of Renal Artery Stenosis: Presentation, Diagnosis, and Management Options in Secondary Hypertension

**DOI:** 10.7759/cureus.92945

**Published:** 2025-09-22

**Authors:** Manvitha Bendagiri Matam, Shalvin Chand, Samyuktha Harikrishnan, Sanathanan Neelakantan Ramaswamy, Nehal K Bhatt, Yashasvi Agarwal, Lubna Mohammed

**Affiliations:** 1 Renal Medicine, St Helier Hospital, London, GBR; 2 General Medicine, Umanand Prasad School of Medicine, University of Fiji, Lautoka, FJI; 3 Research, California Institute of Behavioral Neurosciences & Psychology, Fairfield, USA; 4 General Medicine, Gulf Medical University, Ajman, ARE; 5 Internal Medicine, Government Erode Medical College and Hospital, Erode, IND; 6 Internal Medicine, PramukhSwami Medical College, Karamsad, IND; 7 Internal Medicine, Sir H. N. Reliance Foundation Hospital and Research Centre, Mumbai, IND; 8 Internal Medicine, Jawaharlal Nehru Medical College, Belagavi, IND; 9 Internal Medicine, Dr V.R.K Women's Medical College, Hyderabad, IND

**Keywords:** atherosclerosis, fibromuscular dysplasia, investigations and diagnosis, medical management, renal angioplasty, renal artery stenosis, revascularization, secondary hypertension

## Abstract

Renal artery stenosis (RAS) remains a critical and often underdiagnosed cause of secondary hypertension due to its insidious progression. This traditional review examines the epidemiology, various presentations of RAS, pathophysiology, and diagnostic modalities, with a focus on treatment options based on selected clinical trials.

The prevalence of RAS is notably higher in individuals with systemic atherosclerotic conditions, making atherosclerosis the most common cause of RAS. Less frequently, fibromuscular dysplasia may be involved. Although RAS presents as hypertension in most cases, some people also present with acute syndromes like fluid overload in the lungs and renal insufficiency.

Laboratory investigations, together with imaging modalities, help us diagnose RAS more accurately. Laboratory investigations also aid in confirming renal dysfunction and serologies to identify the underlying causes, such as vasculitis. Imaging modalities like digital subtraction angiography, computed tomography angiography, and magnetic resonance angiography have higher specificity and sensitivity in detecting the RAS.

Although there is an ongoing debate regarding the management of RAS, medical management remains the first-line treatment for renovascular hypertension. Multiple randomized controlled trials have shown that combined revascularization and medical therapy may not significantly improve outcomes in the majority of cases.

This literature review highlights the importance of individualized, evidence-based management of RAS.

## Introduction and background

Renal artery stenosis (RAS) is the blockage of one or more renal arteries supplying the kidney(s) [1]. It is called hemodynamically significant RAS when >60% of the artery is narrowed [2]. Approximately 90% of the RAS is because of atherosclerosis, and the rest 10% is due to fibromuscular dysplasia (FMD) and other rare causes [3].

In RAS, as there is reduced blood flow to the kidneys, it triggers a cascade of events, eventually activating the renin angiotensin aldosterone system (RAAS), leading to secondary hypertension (renovascular hypertension) and further causing renal ischemia [2]. Atheromatous disease and FMD are the most prevalent aetiologies of renovascular hypertension [4], and the most common presentation of renal FMD is resistant hypertension, specifically seen in young women [5]. In one of the studies, the rare causes of renovascular hypertension are described as stenotic and non-stenotic lesions for a better understanding, as addressing those rare causes is also important for the prognosis and management. Stenotic causes include neurofibromatosis type 1, dissection, and arteritis, and non-stenotic causes include aneurysms and arteriovenous fistula [6]. FMD is a rare systemic vascular disease that can affect any arterial bed in the whole body but can specifically involve renal or carotid arteries. Therefore, there is presentation of hypertension or stroke in young women [5].

RAS can present as treatment-resistant hypertension, young age onset hypertension, or even sudden worsening of the primary Hypertension. It can sometimes also present as unexplained hypokalaemia and a rise in Blood Urea Nitrogen [7]. RAS can present in different clinical pictures, as said above, while it can also be asymptomatic and incidentally found on renal imaging [8]. Especially, atherosclerotic renovascular disease (ARVD) can be asymptomatic and have a chronic course undetected for many years [9]. RAS can be diagnosed by Doppler ultrasound (DUS), computed tomography (CT), CT angiogram, magnetic resonance (MR) Angiogram, and digital subtraction angiography (DSA) [4].

Although the pathophysiology of RAS has been known for over 100 years, the accurate treatment options have remained controversial [3]. In a few randomized controlled trials, revascularization surgeries have not been found to have any benefit when used as an adjunct to the optimal medical therapies for RAS [10]. High-risk presentations of ARVD are found to have successful restoration of blood flow after revascularization, which indeed preserved the kidney functions [11]. However, clinicians need to identify the patients who can benefit from revascularization surgery through risk stratification [12]. Generalized renal ischemia, atrophy of the affected kidney, the acute decline in kidney functions, a low resistive index, and low proteinuria are a few factors found to be associated with improved or preserved kidney functions after Revascularization [12].

The purpose of this study is to find out the levels of treatment we could offer patients with various presentations. Do all the patients with secondary hypertension due to RAS end up having revascularization?

Search strategy

This traditional review encompasses 29 articles published between 2008 and 2025. These articles encompass a range of study types, including literature reviews, systematic reviews, randomized controlled trials, and case studies. All articles are available in full text and are published in the English language only. Additionally, we included articles based on human studies and limited the analysis to the adult population only. We excluded articles based on animal studies and those written in languages other than English. Additionally, we excluded studies involving pediatric populations.

PubMed

Renal Artery Stenosis; Secondary Hypertension; Renal Angioplasty; Revascularization; Investigations and diagnosis; Medical Management; Atherosclerosis; Fibromuscular dysplasia

PubMed MeSH

((( "Renal Artery Obstruction/complications"[Mesh] OR "Renal Artery Obstruction/diagnosis"[Mesh] OR "Renal Artery Obstruction/diagnostic imaging"[Mesh] OR "Renal Artery Obstruction/drug therapy"[Mesh] OR "Renal Artery Obstruction/etiology"[Mesh] OR "Renal Artery Obstruction/history"[Mesh] OR "Renal Artery Obstruction/pathology"[Mesh] OR "Renal Artery Obstruction/physiopathology"[Mesh] OR "Renal Artery Obstruction/prevention and control"[Mesh] OR "Renal Artery Obstruction/surgery"[Mesh] )) AND ( "Hypertension, Renovascular/diagnosis"[Mesh] OR "Hypertension, Renovascular/diagnostic imaging"[Mesh] OR "Hypertension, Renovascular/drug therapy"[Mesh] OR "Hypertension, Renovascular/pathology"[Mesh] OR "Hypertension, Renovascular/physiopathology"[Mesh] OR "Hypertension, Renovascular/surgery"[Mesh] )) AND ( "Hypertension, Renal/blood"[Mesh] OR "Hypertension, Renal/diagnosis"[Mesh] OR "Hypertension, Renal/drug therapy"[Mesh] OR "Hypertension, Renal/history"[Mesh] OR "Hypertension, Renal/surgery"[Mesh] )

## Review

Epidemiology

**Figure 1:**
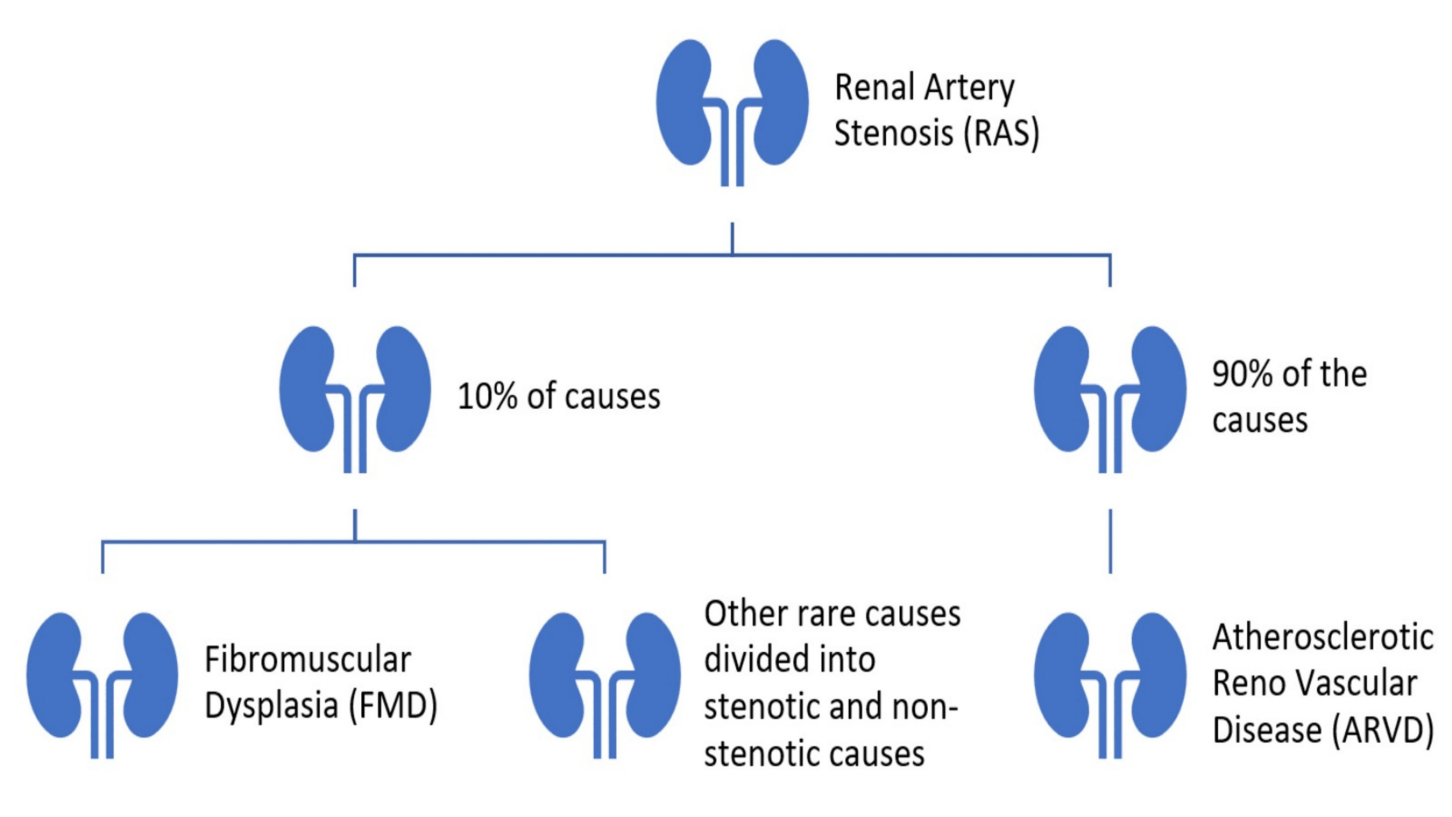
Renal artery stenosis and its causes. Created by the authors based on the data adapted from published source [3]

RAS is a potential cause of secondary hypertension and progressive renal damage. ARVD is the most common cause of RAS, which accounts for 90% of the cases, and the remaining 10% of the cases are due to fibromuscular dysplasia and other rare causes [3]. Figure 1 illustrates the proportional distribution of RAS aetiologies.

ARVD typically affects those people, especially those who are known to have coronary artery disease, peripheral vascular disease, diabetes, and hypertension. A few studies show that the prevalence of ARVD ranged between 12 and 45% in patients with peripheral artery disease and 5 and 30% in patients with coronary artery disease [3]. In patients with mild hypertension, ARVD is expected to be present in 1% of the population, whereas in those with severe hypertension, it goes up to 14-24% (Table 1) [3].

**Table 1:** Prevalence of ARVD in the following co-morbid conditions. Created by the authors based on data from [3] ARVD: Atherosclerotic Renovascular Disease

Comorbidities	Prevalence of ARVD
Peripheral Vascular Disease	12-45%
Coronary Artery Disease	5-30%
Mild Hypertension	1%
Severe Hypertension	14-24%

FMD, on the other hand, is a non-atherosclerotic, non-inflammatory condition seen in young women [5]. ARVD and FMD may exist together in elderly people, making it difficult to diagnose and manage [2]. Due to the nature of the atherosclerotic disease and its insidious progression, RAS is often underdiagnosed, especially in the elderly population with essential hypertension. Acute presentations like flash pulmonary edema and rapid decline in kidney function bring this to light. In the asymptomatic population, 6.8% of the individuals over the age of 65 years have 50% or greater RAS on a duplex ultrasound scan [9].

The prevalence of coronary artery disease in patients with atherosclerotic renal artery stenosis (ARAS) is estimated to fall between 11.3 and 39%. ARAS is the cause of end-stage renal disease in 10-20% of the individuals who are undergoing dialysis [10]. Studies done on ARAS show that stenosis worsens in 51% of instances, which progresses to complete occlusion in 8-16% of cases. Studies have also shown that unilateral ARAS becomes bilateral ARAS in 17% of cases over five years [4]. Other studies also show that there is a significant disease progression in ARAS by 50% by the end of five years [10]. People get renal artery stenosis post-renal transplant, and it is assumed to be one of the most common vascular complications after a renal transplant. Its reported incidence is 8.3 cases per 1000 patient-years [13].

Pathophysiology

Pathophysiology of Hypertension

RAS is the blockage of one or more renal arteries (due to atherosclerosis/ FMD), leading to renal hypoperfusion. Hypoperfusion indeed triggers a cascade of events, hormonally and hemodynamically, by activating the RAAS. This results in hypertension and renal dysfunction [4]. Atherosclerotic renovascular disease leads to the accumulation of lipid-laden plaques in the renal arteries, leading to their narrowing, which indeed reduces the blood flow through afferent arterioles, reducing perfusion pressure. This sensitizes the juxtaglomerular apparatus, which releases renin, and renin converts angiotensinogen to angiotensin 1 and, subsequently, to angiotensin 2. This hormone is a potent vasoconstrictor that stimulates the release of aldosterone. Aldosterone indeed causes sodium and water retention, raising blood pressure [4,7].

Difference between Unilateral and Bilateral Renal Artery Stenosis

In unilateral RAS, the contralateral kidney attempts to compensate for the sodium and water balance, but due to the sustained release of aldosterone following RAAS activation, systemic hypertension develops, and structural damage may also be observed in the bilateral kidneys over time [4]. Bilateral RAS causes volume-dependent hypertension and can rapidly deteriorate kidney functions and potentially cause flash pulmonary edema [3].

Pathophysiology of Chronic Renal Insufficiency

Over a period, RAAS activation causes chronic changes in the systemic vasculature, the heart, and the kidneys. An increase in RAAS activity causes arterial remodeling, inflammation, and oxidative stress, finally leading to kidney fibrosis [3]. Although the renal blood flow is reduced by 30-40%, intrarenal oxygenation will not be affected in the beginning, but the prolonged reduction of blood flow causes irreversible fibrosis in the kidneys [14]. In addition, angiotensin 2 and aldosterone affect gene regulation in cardiac myocytes and fibroblasts, leading to excess inflammation, myocardial hypertrophy, and myocardial fibrosis [3].

Pathophysiology of Fibromuscular Dysplasia Involving the Renal Artery

FMD is a systemic vascular disease that affects small and medium-sized arteries anywhere in the body, and 58% of the cases involve renal arteries. The most common type of FMD is medial fibroplasia, and it causes loss of the elastic membrane but preserves the internal elastic lamina. These changes, alternating with collagen ridges, give a classical “String of beads” appearance on CT angiography (CTA), representing the regions of stenosis and aneurysmal changes. Renal FMD also leads to secondary RAAS activation [5].

A few studies found that the presence of accessory renal arteries (ARAs) correlates with high blood pressure [15]. ARAs are extra arteries that supply the renal hilum and are known as supernumerary renal arteries, and their increased presence is found in hypertensive patients than in normotensive patients [16].

Presentation

Presentation of ARAS

Renovascular hypertension can be seen when there is hemodynamically significant renal artery stenosis [4]. The hypertension that comes with RAS gets refractory over a period of time, and the patient may eventually end up developing ischemic nephropathy, with a progressive reduction in the glomerular filtration rate. Even with three different anti-hypertensives, it can be difficult to bring down the blood pressure [7]. Therefore, it can present as a hypertensive crisis with acute deterioration of kidney function [17]. 

It can also present as a sudden rise in serum creatinine by 30% above its baseline after initiation of angiotensin receptor blockers/angiotensin convertase enzyme inhibitors or as a deranged kidney function after endovascular aortic stenting. Additionally, people develop abdominal and flank bruit with renal artery stenosis [7].

It is not practical to categorize patients with atherosclerotic renal artery stenosis as symptomatic or asymptomatic, as the symptoms are often delayed until there is an end-organ insult and AKI occurs. Until then, the disease mostly remains silent [4]. Patients can present as unexplained flash pulmonary edema requiring recurrent admissions to the hospital as decompensated heart failure, but echo shows normal left ventricular function [3,18].

Presentation of Renal FMD

FMD primarily involves renal arteries and cranio-cervical arteries. It should be suspected in young women with unexplained hypertension, as this is the most common presentation of it. It can present as flank pain, hematuria, and unexplained hypokalemia due to the activation of RAAS. In young patients with a history of stroke and the above-mentioned symptoms, RAS should be considered. Systemic findings may include abdominal or carotid bruit depending on the vascular territories affected [5].

Presentation of RAS in Transplant Kidneys

RAS can also occur in post-transplant patients between three months and two years after the surgery. Some cases may present as early as within one week of the transplant surgery. The common presentation would be treatment-resistant hypertension with or without graft dysfunction [13].

Difference between Presentations of Unilateral RAS and Bilateral RAS

In unilateral RAS, the primary mechanism involves activation of the RAAS by the affected kidney, leading to systemic hypertension. The contralateral kidney responds with pressure diuresis, but sustained high pressure can cause hyperfiltration and chronic damage, resulting in proteinuria. As the disease progresses, nephrotic-range proteinuria and worsening hypertension may develop, both of which are associated with a poor prognosis [3,4].

In bilateral RAS, or significant RAS in a solitary functioning kidney, the primary mechanism involves systemic activation of the RAAS but with no functional kidney to compensate. Persistent glomerular hypoperfusion can cause irreversible renal damage and interstitial fibrosis, ultimately leading to diminished diuresis. This leads to sodium and water retention, resulting in severe hypertension and fluid overload. Patients often present with acute kidney injury and recurrent episodes of flash pulmonary edema, a condition known as "Pickering syndrome" [3,4].

Investigations

Laboratory Studies

The following laboratory investigations help us assess the degree of renal dysfunction and, therefore, help us manage the condition.

Serum creatinine levels, 24-hour urine collection for protein, and protein-creatinine ratio from a random urine sample [7]. Other tests like serology for conditions such as systemic lupus erythematosus or vasculitis, including antinuclear antibodies (ANA), complement levels (C3, C4), and antineutrophil cytoplasmic antibodies (ANCA) help us in diagnosing the cause [7].

Diagnostic Laboratory Investigations

The plasma renin activity (PRA) test can be used to determine whether individuals with ARAS have responded to the revascularization procedure or not. As it can be affected by various parameters like sex, age, race, renal disease, and other comorbidities, its sensitivity is only 57% while the specificity is 66% [10].

Captopril renography works by inhibiting angiotensin converting enzyme and subsequently measuring glomerular filtration rates in both the kidneys - one unaffected and the other affected by ARAS. It allows for the measurement of plasma aldosterone concentration (PAC), plasma renin concentration (PRC), and PRA. A PAC/ARC ratio greater than 40 or a PAC/PRA ratio greater than 200 is indicative of renovascular hypertension. Although this test appears to be reliable, it has its own limitations. It is not effective in patients with bilateral kidney disease or poor renal function and cannot localize the site of stenosis. Therefore, this investigation has a sensitivity of 74% while the specificity is 59% [10].

Bilateral renal vein renin assay is the ratio between the renin level of the ischemic kidney and that of the contralateral kidney. These measurements aid in selecting the individuals with hypertension and ARAS who may benefit from interventions. In the majority of cases, these individuals respond to the treatment. However, it has its own limitations as it cannot differentiate people with essential hypertension from those with ARAS. Therefore, this test is associated with a high rate of false positives and false negatives, resulting in both sensitivity and specificity of only 67% [10].

Imaging Modalities

Doppler ultrasound: DUS is an effective tool for identifying ARAS and evaluating its extent by directly imaging the renal arteries and analyzing blood flow velocity. A peak systolic velocity exceeding 200 cm/s typically suggests stenosis greater than 50%, with reported sensitivity and specificity rates of approximately 95% and 90%, respectively [7].

Due to its affordability and non-invasive approach, duplex ultrasound is commonly used to screen for ARVD. However, its effectiveness can be influenced by the technician’s expertise and may be compromised in individuals with obesity or intestinal gas [3,19]. This test also remains the first-line investigation in identifying fibromuscular dysplasia, but has the same limitations [5].

CT angiography: CTA provides detailed imaging with excellent spatial resolution and fast scan times. It is highly accurate for identifying renal artery stenosis over 50%, with a reported sensitivity of 94% and specificity of 93% [10]. It also helps in identifying ARAs [7]. It produces the classic "string-of-beads" appearance on imaging, which is diagnostic of fibromuscular dysplasia [5]. It is highly valuable for monitoring stents and for assessing patency during follow-up after bypass surgeries [19].

Magnetic resonance angiography (MRA): It offers non-invasive visualization of the renal arteries without the use of ionizing radiation. In addition to anatomical imaging, it provides functional data by detecting hemodynamic disturbances, such as increased blood flow turbulence. MRA is also valuable in the assessment of in-stent restenosis. Studies have reported a sensitivity of 96% and a specificity of 92% for detecting renal artery stenosis of 50% or greater [10].

Gadolinium-enhanced magnetic resonance angiography (Gadolinium-enhanced MRA): It has been shown to offer superior sensitivity and specificity compared to non-contrast MRA techniques for detecting renal artery stenosis. While earlier concerns about the development of nephrogenic systemic fibrosis (NSF) limited the use of gadolinium-based contrast agents in patients with significantly reduced renal function (GFR <30 mL/min/1.73 m²), recent evidence supports the safety of newer group II gadolinium agents in individuals with chronic kidney disease (CKD). These agents have demonstrated a low risk of NSF, thereby expanding the clinical utility of contrast-enhanced MRA in high-risk populations [18].

Conventional angiography: Conventional angiography remains the gold standard investigation for the identification of renal artery stenosis [7,19]. There are several types of conventional angiography, including conventional aortography, intravenous subtraction angiography, intra-arterial subtraction angiography, and carbon dioxide angiography (for patients who are unable to receive iodinated contrast) [7]. Conventional aortography produces excellent images of the renal arteries, but it requires an arterial puncture, which carries risks such as embolism, bleeding, and contrast-induced acute tubular necrosis [7]. The stenosis involving more than 70% of the vessel diameter is classified as severe, while moderate to severe stenosis ranges from 50% to 70% [19].

Management

Management of Fibromuscular Dysplasia

Medical and lifestyle management: Antiplatelet therapy should be initiated in any asymptomatic patient diagnosed with FMD. It is particularly recommended for patients with a history of stroke or renal infarction. Effective blood pressure control remains the mainstay of FMD management. Angiotensin 2 convertase enzyme (ACE) inhibitors and angiotensin receptor blockers (ARBs) are considered first-line agents, but renal function should be closely monitored during treatment. Other antihypertensive medications, such as diuretics, beta-blockers, and calcium channel blockers, may also be used as needed. Lifestyle modifications, particularly smoking cessation, are critically important in the management of FMD [5].

Surgical Management

Revascularization may be considered for patients with resistant hypertension. The main goal of this approach is to prevent ischemic nephropathy. Percutaneous transluminal angioplasty remains the first-line surgical intervention and has been shown to improve the condition in 60%-80% of cases. Angioplasty and stent placement can be considered when there is dissection or perforation due to FMD. One of the meta-analyses reports a 45.7% improvement in blood pressure, but when including the population who were already on anti-hypertensives, the cure rate has decreased to 35.8% [5].

Medical Management of ARAS

Any patient who is newly diagnosed with ARAS must undergo strict lifestyle modifications as the first step of management alongside medical therapy. Diet, smoking cessation, and physical activity complement the medical management [10].

Hypertension in patients with RAS can be controlled in 90% of cases with medical therapy alone. However, Atherosclerotic renal artery stenosis is the most common disease for which treatment remains uncertain, and there is still room for research [19]. Medical management of ARVD should aim to reduce cardiovascular risk and maintain kidney function by lowering hypertension [3]. ACE inhibitors and ARBs are the first-line medical therapies that we should use in these patients to control hypertension [3]. They are also considered the first-line treatment options in patients with ARA-related hypertension [15].

It becomes challenging to treat patients with atherosclerotic renovascular disease in a single kidney, bilateral arterial involvement, in a transplant kidney, and especially when patients present with recurrent cardiac destabilization syndromes and malignant hypertension [19]. Although ACE inhibitors and ARBs help with hypertension, they need to be used carefully as they can cause a decline in kidney function acutely. A >30% decline in the eGFR (estimated glomerular filtration rate) should raise a concern, and we should be thinking about other options like revascularization [19,20]. In terms of treating hypertension related to renovascular disease, general guidelines for using calcium channel blockers, beta-blockers, and statins to lower lipid levels can also be considered [3].

One of the articles mentioned optimal medical therapy [19]. “Optimal Medical therapy” essentially comprises anti-hypertensives, as said above, antiplatelets, and statins, along with maintaining a good lifestyle, diet, and good glycaemic control. However, there is always a concern about the fatal impact of low blood pressure on the kidneys by using anti-hypertensives, as it can lead to ischemic insult of renal parenchyma [19]. Some studies showed that statins are capable of modifying the kidney’s microvascular milieu, controlling inflammatory changes, and eventually limiting fibrosis [21]. Moreover, statins and other lipid-lowering agents were found to significantly reduce morbidity and mortality in people with progressive renal disease. A retrospective study compared the overall survival and renal survival over a period of 11 years in 104 patients with ARVD. Among them, 68 received statins because of the elevated lipid levels, and 36 did not as their lipid levels were normal. The study found that statin therapy was associated with a lower rate of progression of renal insufficiency and reduced overall mortality, with 7.4% of patients who received statins reaching renal end point, compared to 38.9% in the non-statin group [22]. Other observational studies have shown that the use of beta-blockers and low-dose aspirin has given similar outcomes of reduced mortality risk in patients with ARVD [23].

Surgical Management of ARAS

Failure of optimal medical therapy may advise us to decide whether revascularization is a potential option to be considered [24]. The journey of evidence in revascularization for ARVD has unfolded through a distinctive transformation [11]. In the early 1990s, revascularization was found to be very interesting because of significant reductions in blood pressure and stable kidney function post-surgery [25].

Percutaneous transluminal renal arterial stenting (PTRAS) is one of the revascularization procedures available for patients with hemodynamically significant renal artery stenosis [26]. One of the studies showed that out of 265 patients with ARVD (> or = 50% stenosis) who were treated with percutaneous transluminal renal angioplasty plus stenting, there was significant improvement in the blood pressures in the median follow-up of 23.8months post-surgery. Among them, 53.9% of the patients also showed improvement in the estimated glomerular filtration rate (eGFR), whereas 15.5% of the patients' eGFR remained unchanged, and 30.6% of the patients' eGFR continued to deteriorate [27]. Patients whose eGFR or BP (blood pressure) improved or stabilized post-surgery had low pre-procedural BP or severe stenosis when compared to people with declining kidney function post-surgery [27].

One of the studies explained that patients with controlled BP and stable renal function are unlikely to benefit from PTRAS [24]. Another study also added that patients with chronic kidney disease stage-5 (CKD-5), who are receiving hemodialysis for more than three months or pole-to-pole kidney size of <7cms (severely low renal functions) are unlikely to get any benefit from revascularization [28]. 

Revascularization can be done through endovascular procedures or open surgery, but open surgeries are mostly limited to complex anatomical lesions [19]. Although we do endovascular stenting for ARAS, in 10-20% of the cases, there would be restenosis [19]. A few studies advise that renovascular hypertension related to the ARA may be treated with renal denervation [15,16]. In one of the studies where five patients with ARA and resistant hypertension underwent renal denervation, four people were found to have reduced BP after this. People with ARA and resistant hypertension have more benefits in denervation than revascularization [15].

Evidence from the controlled trials

The DRASTIC (Dutch Renal Artery Stenosis) study suggested that among 106 patients with ARVD, a few of them were given medical therapy, and a few of them were offered revascularization surgery. At the follow-up review at three months and 12 months, there was no significant difference in the BP levels between these two groups. However, over half of the patients who were given medical treatment ended up having revascularization for uncontrolled BP (with three or more anti-hypertensives) and declining kidney function [3].

The CORAL (Cardiovascular Outcomes in Renal Atherosclerotic Lesions) Trial suggested that renal artery stenosis had similar clinical outcomes when given optimal medical therapy alone and when optimal medical therapy plus renal artery stenting was done together [19,29]. The ASTRAL (The Angioplasty and Stenting for Renal Atherosclerotic Lesions) trial also shows no overall benefit of renal Revascularization to renal and cardiovascular outcomes when compared with medical therapy alone [19]. 

The following table summarizes the above-explained randomized controlled trials (Table 2) [3,19,29].

**Table 2:** Summary of the randomized controlled trials and their outcomes. Created by the authors based on data from [3,19,29] DRASTIC: Dutch Renal Artery Stenosis Trial; CORAL: Cardiovascular Outcomes in Renal Atherosclerotic Lesions Trial; ASTRAL: The Angioplasty and Stenting for Renal Atherosclerotic Lesions.

Randomized Controlled Trials	Inference of the Trials
DRASTIC	No difference in the blood pressures between people who received medical therapy and revascularization therapy at the 3-monthly and 12-monthly follow up, but over half of the population who were given medical therapy ended up having revascularization later for uncontrolled Blood pressures
CORAL	Similar clinical outcomes when given optimal medical therapy alone and when optimal medical therapy plus renal artery stenting was done together
ASTRAL	No overall benefit of renal Revascularization to renal and cardiovascular outcomes when compared with medical therapy alone

## Conclusions

Atherosclerotic renovascular disease is the leading cause of RAS and is strongly associated with co-morbid conditions like coronary artery disease, peripheral vascular disease, diabetes, and hypertension. RAS is challenging to diagnose due to its insidious progression and remains silent until it eventually needs clinical attention. Therefore, timely diagnosis is important. Lifestyle modifications such as smoking cessation, diet management, and exercise should be emphasized along with other management options. Optimal medical therapy remains the first-line treatment for RAS in most cases. ACE inhibitors and ARBs are the first-line drugs to manage renal FMD and ARAS. Statins, antiplatelets, and beta blockers are proven to improve the prognosis of the RAS. Any patients with FMD must be started on antiplatelet therapy. 

Patients with atherosclerotic renovascular disease in a single kidney, bilateral arterial involvement, in a transplant kidney, in patients with recurrent cardiac destabilization syndromes, and malignant hypertension, ACE inhibitors and ARBs are still considered the first-line treatment options, but they need to be used carefully as they can cause a decline in kidney function acutely. This should help us consider revascularization. While observational studies show potential benefits of revascularization procedures, large randomized trials (DRASTIC, ASTRAL, CORAL) have not demonstrated any consistent advantages over medical therapy alone. Revascularization has a significant role in selected patients. Individuals with hemodynamically significant RAS, resistant hypertension, recurrent cardiac destabilization syndrome, progressive renal dysfunction, and complex anatomical lesions causing RAS are likely to benefit from revascularization. Revascularization is unlikely to be beneficial or is not indicated for those with controlled hypertension, stable renal function, severely low renal function, or those who have atrophied kidneys. Treatment decisions should be individualized, as not all patients benefit from revascularization.
